# Multi-omics insights into potential mechanism of SGLT2 inhibitors cardiovascular benefit in diabetic cardiomyopathy

**DOI:** 10.3389/fcvm.2022.999254

**Published:** 2022-10-05

**Authors:** Yangbo Xi, Dongping Chen, Zhihui Dong, Jinhua Zhang, Hingcheung Lam, Jiading He, Keyi Du, Can Chen, Jun Guo, Jianmin Xiao

**Affiliations:** ^1^The First Clinical Medical College, Jinan University, Guangzhou, China; ^2^Department of Cardiology, The First Affiliated Hospital of Jinan University, Guangzhou, China; ^3^Central Laboratory, Binhaiwan Central Hospital of Dongguan, The Dongguan Affiliated Hospital of Jinan University, Dongguan, China; ^4^Department of Pathology, Binhaiwan Central Hospital of Dongguan, The Dongguan Affiliated Hospital of Jinan University, Dongguan, China; ^5^Department of Cardiology, Binhaiwan Central Hospital of Dongguan, The Dongguan Affiliated Hospital of Jinan University, Dongguan, China

**Keywords:** diabetic cardiomyopathy, SGLT2 inhibitors, mechanisms, proteomics, metabolomics

## Abstract

**Background:**

Metabolic and energy disorders are considered central to the etiology of diabetic cardiomyopathy (DCM). Sodium-glucose cotransporter-2 inhibitors (SGLT2i) can effectively reduce the risk of cardiovascular death and heart failure in patients with DCM. However, the underlying mechanism has not been elucidated.

**Methods:**

We established a DCM rat model followed by treatment with empagliflozin (EMPA) for 12 weeks. Echocardiography, blood tests, histopathology, and transmission electron microscopy (TEM) were used to evaluate the phenotypic characteristics of the rats. The proteomics and metabolomics of the myocardium in the rat model were performed to identify the potential targets and signaling pathways associated with the cardiovascular benefit of SGLT2i.

**Results:**

The diabetic rat showed pronounced DCM characterized by mitochondrial pleomorphic, impaired lipid metabolism, myocardial fibrosis, and associated diastolic and systolic functional impairments in the heart. To some extent, these changes were ameliorated after treatment with EMPA. A total of 43 proteins and 34 metabolites were identified as targets in the myocardium of diabetic rats treated with EMPA. The KEGG analysis showed that arachidonic acid is associated with the maximum number of related pathways and may be a potential target of EMPA treatment. Fatty acid (FA) metabolism was enhanced in diabetic hearts, and the perturbation of biosynthesis of unsaturated FAs and arachidonic acid metabolism was a potential enabler for the cardiovascular benefit of EMPA.

**Conclusion:**

SGLT2i ameliorated lipid accumulation and mitochondrial damage in the myocardium of diabetic rats. The metabolomic and proteomic data revealed the potential targets and signaling pathways associated with the cardiovascular benefit of SGLT2i, which provides a valuable resource for the mechanism of SGLT2i.

## Introduction

Diabetes is the most important comorbidity of cardiovascular disease (CVD), and CVD is the main cause of death and disability in patients with diabetes worldwide (1–4). At present, the global prevalence of diabetes is steadily increasing. An estimated 578 million people will be diagnosed with diabetes by 2,030, and the total number of patients will reach 700 million by 2,045 (2). Many studies have demonstrated that diabetes can affect cardiac structure and function independent of coronary artery disease, ischemia, hypertension, and other risk factors, which are recognized as a distinct clinical entity called diabetic cardiomyopathy (DCM) (5, 6). DCM is a common cardiovascular complication in diabetes patients characterized by myocardial fibrosis, ventricular remodeling, and cardiac dysfunction (7, 8). It is closely linked to the high incidence and mortality of heart failure in patients with diabetes (9, 10). Patients with diabetes and even prediabetes [defined as impaired fasting glucose (IFG), impaired glucose tolerance (IGT), or elevated HbA1c] were associated with an increased risk of HF, and DCM plays an important role in this progress (11). Although DCM was identified more than 40 years ago, and many pathogenic mechanisms have been discovered through extensive research, effective strategies for DCM prevention and treatment remain elusive. Sodium-glucose cotransporter-2 inhibitors (SGLT2i) are the first anti-diabetic medications to effectively reduce the risk of cardiovascular death and heart failure in patients with type 2 diabetes. Notably, increasing evidence has demonstrated the beneficial cardiovascular effects of SGLT2i, independent of its glycemic control effects (12, 13).

However, since the main mechanistic contributor to the cardiovascular benefit of SGLT2i has not been elucidated, further studies are required to explore the mechanisms underlying the clinical benefits of SGLT2 inhibitors. Recent research has shown that SGLT2i improves cardiac metabolism by shifting myocardial substrate utilization from glucose toward oxidation of FAs, ketone bodies, and branched-chain amino acids, thereby improving the energy-starved heart in diabetes and heart failure (14, 15). In diabetic hearts, insulin resistance and HG induce metabolic alterations that decrease glucose utilization while increasing FA uptake and β-oxidation (16). The mismatch between uptake and β-oxidation of FAs results in intracellular lipid accumulation and lipotoxicity that initiates a cascade of downstream pathophysiological changes and myocardial mechanics (17). In terms of the DCM mechanism, mitochondrial dysfunction, impairment of mitochondrial Ca^2+^ handling, oxidative stress, increased production of advanced glycation end products (AGEs), inflammation, activation of the renin-angiotensin-aldosterone system (RAAS), autonomic neuropathy, endoplasmic reticulum stress, microvascular dysfunction, cardiomyocyte death, and cardiac metabolic disorders participate in the pathophysiological process (7, 17–19). The interactions between these abnormal pathophysiological processes result in cardiac stiffness, fibrosis, and hypertrophy, leading to diastolic and systolic dysfunction and heart failure.

Although the mechanism of DCM appears to be multifactorial, the pathophysiological changes seem to be induced by cardiac metabolic alterations, which are distinct from other cardiomyopathies (17, 20). While metabolic and energy disorders are considered central to the etiology of DCM and the progression to failure, the metabolic changes associated with functional and eventually cardiac failure remain unclear, especially after being treated with SGLT2i. In the present study, we present an integrative multi-omics analysis, including proteomics and metabolomics, to systematically analyze the metabolic changes in the diabetic heart, through which we could evaluate the progression of metabolic disturbances in the diabetic heart and the potential mechanisms underlying the cardiovascular clinical benefits of SGLT2i.

## Materials and methods

### Animal models

A total of 36 7-week-old male rats were provided by the SPF Biotechnology company (SPF, Beijing, China). The rats were housed in the hygienic animal facility at 20–25°C temperature and 40–70% relative humidity with a 12/12 h light-dark cycle. All animal experiments were conducted per the procedures approved by the Medical Ethics Committee of the Dongguan Affiliated Hospital of Jinan University.

To induce diabetes, the rats were intraperitoneal (i.p.) injected with a single dose of streptozocin (STZ, Macklin, Shanghai, China, S817944, 70 mg/kg, diluted to 1% using fresh sodium citrate buffer, pH = 4.5) for 5 consecutive days. The rats in the control group (*n* = 6) were i.p. injected with equivalent doses of STZ solvent. Once a week, tail-vein blood was collected from the rats to test their fasting blood glucose levels. All animals were fed a normal diet for 18 weeks, and those with blood glucose levels of > 16.7 mmol/L for three consecutive weeks were presumed to be diabetic. The diabetic rats were randomly divided into the hyperglycemia (HG) group (*n* = 10) and the empagliflozin (EMPA) group (*n* = 8), matched for body weights and uniformity. The rats in the EMPA group were treated with empagliflozin (EMPA; Boehringer Ingelheim) for 12 weeks. EMPA was mixed in drinking water at an average dose of 30 mg/kg/day.

### Echocardiography

Animals were intraperitoneally anesthetized with phenobarbital (2%, 50 mg/kg, i.p., H20057384, fujian mindongrejuenation, China). Two-dimensional (2D) guided M-mode echocardiography was performed at 18 and 30 weeks using an L15-7io probe (Ultrasound Transducer Bothell, WA 98021, USA). Left ventricle internal dimension at end-diastole/systole (LVID;d/s), left ventricle posterior wall thickness at end-diastole (LVPW’d), and interventricular septum thickness at end-diastole (IVS’d) were measured by M-mode tracing. The left-ventricular fractional shortening (FS) was calculated as [(LVIDD-LVIDS)/LVIDD] *100%, the ejection fraction (EF) percentage using the equation: [(EDV-ESV)/EDV] *100%, where EDV represents end-diastolic volume and ESV represents end-systolic volume.

### Blood measurements and histopathology

Rats were anesthetized with an intraperitoneal injection of phenobarbital sodium (60–80 mg/kg, i.p.). Blood samples were collected from all the rats. Plasma samples were used for the measurement of the following parameters: blood insulin (CUSABIO, Wuhan, China, E05070r), glucagon (CUSABIO, CSB-E12800r), total cholesterol (TC, Nanjing Jiancheng, China, A111-2-1), triglyceride (TG, Nanjing Jiancheng, A110-1-1), high-density lipoprotein cholesterol (HDL-c, A112-1-1), low-density lipoprotein cholesterol (LDL-c, A113-1-1), brain natriuretic peptide (BNP, CUSABIO, CSB-E07972r), cardiac troponin I (cTn I, CUSABIO, CSB-E08594r), and creatinine (Cr, Nanjing Jiancheng, C011-2-1).

After euthanasia, the heart was harvested and processed for subsequent tissue and molecular analyses. Mid-ventricular heart sections were fixed in 4% paraformaldehyde, embedded in paraffin, sectioned at 4 μm thickness, and stained with hematoxylin and eosin (H&E, BBC biochemical) and Masson (Abcam, UK, ab150681) according to the manufacturer’s protocol. The fibrotic content (three rats per group, 10 fields each) was quantified using ImagePro PLUS software (Media Cybernetics, USA).

### Transmission electron microscopy

The mitochondrial structure was further examined by standard transmission electron microscopy (TEM) in the laboratory of Guangzhou Huiyuanyuan Pharmaceutical Technology Co., LTD. Fresh myocardium sample preparation for TEM was performed as previously described (21).

### Proteomics

The heart sample was ground in liquid nitrogen, lysed with PASP buffer (100 mM NH_4_HCO_3_, 8 M Urea, pH 8), and crushed by ultrasonication on ice for 5 min. The raw data were obtained using the following steps: total protein extraction from the sample, protein quality test, trypsin treatment (22), DDA spectrum library construction, and liquid chromatography-mass spectrometry (LC-MS)/MS analysis-DIA mode (23). The raw file obtained from the DDA scanning was decomposed by Proteome Discoverer 2.2 (PD 2.2, Thermo Fisher Scientific), which converted the spectrum data into protein data. After quality control, the protein data was imported into Spectronaut (Version 14.0, Biognosys) to construct the DDA library. Moreover, the raw files obtained by DIA scanning were compared to the DDA library for protein identification by Spectronaut. The protein quantitation results were statistically analyzed using the *t*-test. Proteins with significantly different quantities between the experimental and control groups (*p* < 0.05 and | log2FC| > 1.5) (fold change, FC) were defined as differentially expressed proteins.

### Metabolomics

**FIGURE 1:**
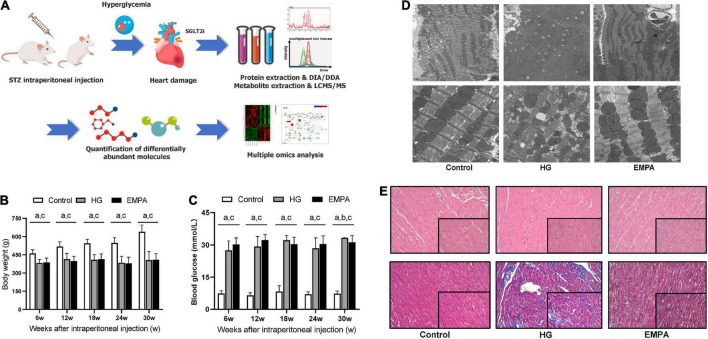
The study design and phenotypic characteristics of the rats after STZ intraperitoneal injection. **(A)** Schematic of the study design. **(B,C)** The body weight **(B)** and blood glucose **(C)** of rats in the control, high glucose (HG), and empagliflozin (EMPA) group. (a) comparison is significant for Control vs. HG, (b) comparisons are significant for HG vs. EMPA, and (c) is significant for Control vs. EMPA. **(D)** Transmission electron microscopy was observed on the rat heart of the three groups. 5,000x (upper part); 20,000x (lower part). **(E)** Representative photomicrograph of H and E staining (upper part) and Masson’s staining (lower part) of the myocardium, 200x (insert 400x).

LC-MS technology based on the ACQUITY UPLC Xevo TQ-S platform was used to perform N300 metabolomics. The experimental process mainly included sample collection, target metabolite extraction, LC-MS/MS on-board, and data analysis (24, 25). The MS detection process relies on on-machine detection of blank samples (blank), quality control samples (QC), and experimental samples. To obtain absolute quantitative results of metabolites in the samples, we performed chromatographic data analysis using MassLynx V4.1 software. Next, multivariate statistical analysis of metabolites, including principal component analysis (PCA), partial least squares discriminant analysis (PLS-DA), etc., revealed the differences in metabolic patterns of different groups. Hierarchical clustering (HCA) and metabolite correlation analysis were used to reveal relationships between sample metabolites. Finally, the biological significance, such as metabolic pathways of the metabolites, was explained by functional analysis. The proteomics and metabolomics were carried out at the laboratory of Beijing Novogene Co., Ltd. The schematic depiction of this study is shown in Figure 1A.

### Statistical analysis

Data were expressed as mean ± SD. One-way ANOVA determined differences between groups with the SPSS 13.0 software and GraphPad Prism 6.0. For all the tests, a two-sided *p*-value of < 0.05 was considered significant.

## Results

### Phenotypic characteristics of the animals

Compared to the rats in the control group, STZ intraperitoneal injection led to marked HG (blood glucose levels > 16.7 mmol/L) and weight loss in rats. These significant weight losses and HG were observed at 1 week and persisted for up to 30 weeks (Figures 1B,C). During the follow-up, there was no significant weight gain in rats fed with EMPA. At 30 weeks (EMPA fed for 12 weeks), the blood glucose of rats was reduced (*p* < 0.05) but remained at a high level (> 30.0 mmol/L). The plasma parameters of TG, TC, and HDL-c were increased in the EMPA group compared with the control group (*p* < 0.05), but no significant changes were detected in LDL-c, BUN, Cr, BNP, insulin, and glucagon among the groups at 30 weeks (Table 1).

**TABLE 1:** Plasma parameters of animals.

	Control (*n* = 6)	HG (*n* = 10)	EMPA (*n* = 8)	*P*-value (ANOVA)
TG, mmol/L	1.42 ± 0.49^a^	2.15 ± 0.85	2.58 ± 0.96	0.052
TC, mmol/L	1.57 ± 0.36^a^	1.69 ± 0.55^b^	2.33 ± 0.73	0.046
HDL-c, mmol/L	0.30 ± 0.12^ac^	0.53 ± 0.24	0.58 ± 0.18	0.040
LDL-c, mmol/L	1.08 ± 0.53	0.87 ± 0.62	1.07 ± 0.58	0.707
BUN, pg/ml	0.46 ± 0.28	1.32 ± 0.83	1.69 ± 1.63	0.135
Cr, μmol/L	44.87 ± 10.18	42.62 ± 5.91	43.67 ± 10.53	0.882
BNP, ng/ml	<0.125*	0.31 ± 0.72	0.81 ± 0.97	0.166
Insulin, μUI/ml	0.65 ± 0.19	0.63 ± 0.24	0.58 ± 0.25	0.844
Glucagon, pg/ml	39.92 ± 11.81	36.16 ± 23.96	31.09 ± 12.97	0.667

HG, hyperglycemia; EMPA, empagliflozin; TG, triglyceride; TC, total cholesterol; HDL-c, high-density lipoprotein cholesterol; LDL-c, low-density lipoprotein cholesterol; BUN, blood urea nitrogen; Cr, creatinine; BNP, brain natriuretic peptide. With LSD multiple comparison tests, (a) comparison significant for control vs. EMPA, (b) comparisons significant for HG vs. EMPA, and (c) comparisons significant for control vs. HG. *Below the detection limit of the kit. Data were presented as mean ± standard deviation.

**FIGURE 2:**
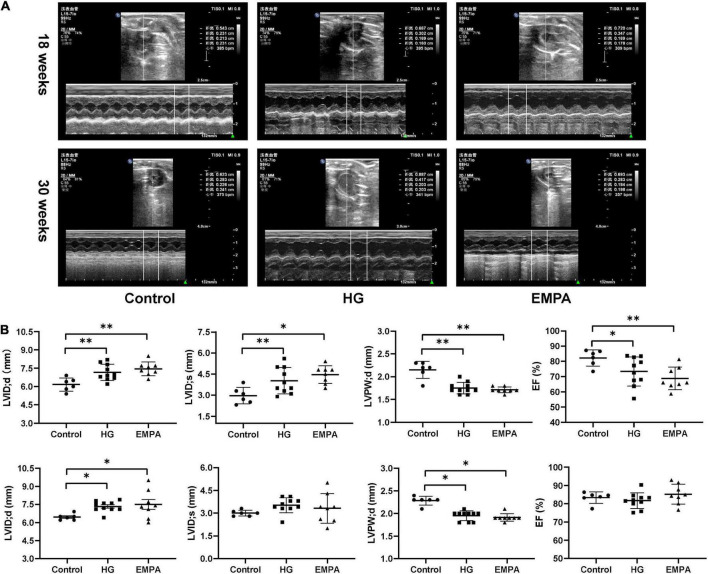
In three groups, echocardiographic measurements of the rats at 18 weeks and 30 weeks after STZ injection. **(A)** Representative M-mode tracings of rats in three groups (*n* = 6 for the control group, *n* = 10 for the high glucose (HG) group, and *n* = 8 for the empagliflozin (EMPA) group). **(B)** Plots of echocardiographic measurement results for left ventricle internal dimension at end-diastole/systole (LVID;d/s), left ventricle posterior wall thickness at end-diastole (LVPW’d), and ejection fraction (EF) percentage, upper part for 18 weeks, the lower part for 30 weeks. *p < 0.05, ^**^p < 0.01.

TEM imaging of myocardium in the HG group at 30 weeks revealed pleomorphic mitochondria, lipid deposition, and fewer cardiomyocytes, indicating mitochondrial damage and impaired lipid metabolism in cardiomyocytes (Figure 1D). Masson’s staining showed significantly increased myocardial fibrosis in the HG group (Figure 1E). Notably, lipid deposition, mitochondrial changes, and myocardial fibrosis were ameliorated in the EMPA group. In addition, echocardiography showed abnormalities in cardiac structure and movement in the STZ-treated rats, which manifested as increased left ventricle internal dimension at end-diastole/systole (LVID; d/s), left ventricle posterior wall thickness at end-diastole (LVPW; d), and decreased EF (Figure 2).

### Overview of proteome differences

**FIGURE 3:**
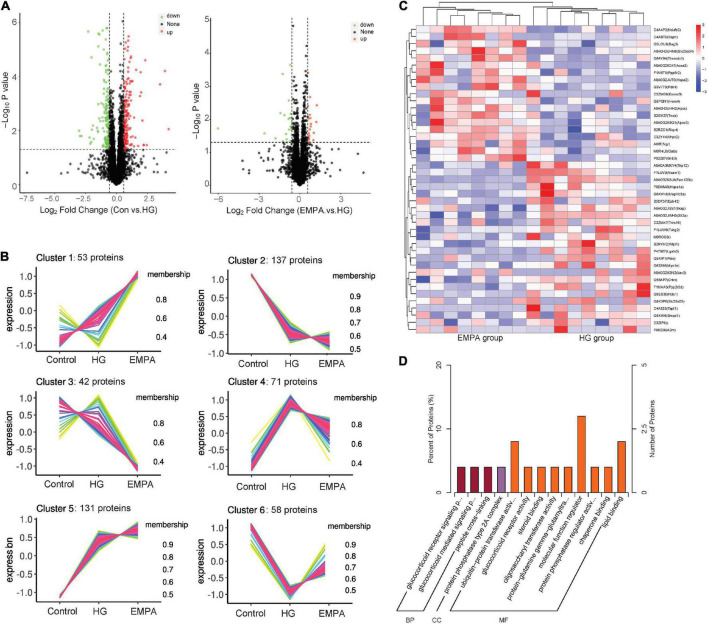
The protein expression changes in the myocardium. **(A)** Volcano plots of protein expression changes. Cut-off value: *p* < 0.05, fold change (FC) ≥ 1.5 (upregulation) or ≤ 0.67 (downregulation), the red and green point represents the upregulated and downregulated proteins, respectively. **(B)** C-means cluster diagram of differential proteins. The proteins are classified into 6 clusters according to their expression levels in each group, and the horizontal coordinate is the group, the vertical coordinate is the protein expression level (correction *Z*-value), each line represents one protein, larger membership values indicate that the protein is closer to the average level protein of this cluster. **(C)** Hierarchical clustering analysis of differential in empagliflozin (EMPA) and high glucose (HG) group. **(D)** GO enrichment of differential proteins in EMPA and HG groups. BP, biological process; CC, cellular component; and MF, molecular function.

We performed protein expression profiling of rat hearts from the three groups at 30 weeks (Figure 3). A total of 4,452 proteins were identified (Supplementary Table 1). Of these, 492 proteins exhibited differential expression between at least two groups (*p* < 0.05, fold change > 1.5 or < 0.67). The C-means cluster analysis revealed that treatment with EMPA could reverse some dysregulated proteins caused by STZ-induced diabetes in the rat’s myocardium (Figure 3B). The HCA analysis (Figure 3C) revealed that the regulatory profiles of proteins differed significantly between the HG and EMPA groups (upregulated for 185 proteins, downregulated for 162 proteins). GO enrichment analysis showed that the upregulated proteins of the EMPA group (compared with the HG group) were mostly associated with the regulation of the glucocorticoid signaling pathway (GO: 0042921, GO: 43402, GO: 0004883) and lipid metabolic processes such as lipid transport (GO: 0006869), lipoprotein metabolic process (GO: 0042157), and lipid binding (GO: 0008289), while the downregulated proteins were associated with MF regulator (GO: 0098772), enzyme regulator activity (GO: 0030234), and peptide cross-linking (GO: 0018149) (Figure 3D). In the category of cellular component (CC), the upregulated and downregulated proteins were enriched in mitochondrial respiratory chain complex I (GO: 0005747) and protein phosphatase type 2A complex (GO: 0000159), respectively. Solute carrier family 25 member 34 (SLC25A34), serum amyloid P-component (APCS), and probable E3 ubiquitin-protein ligase HERC4 were the three most significantly upregulated proteins in the EMPA group compared with the HG group, which were associated with fatty acid (FA) metabolism, mitochondrial function, and apoptosis. Its upregulation may explain why EMPA could alter lipid deposition, mitochondrial changes, and myocardial fibrosis in the diabetic heart.

Among the differential proteins of the control group and the HG group, GO enrichment showed that most of the upregulated proteins were associated with FA and lipid metabolic processes such as monocarboxylic acid metabolic process (GO: 0032787), cellular lipid metabolic process (GO:0044255), lipoprotein metabolic process (GO: 0042157), FA beta-oxidation (GO: 0006635), and lipid transport (GO: 0006869), while the downregulated proteins were associated with oxidation-reduction process (GO: 0055114), GDP-mannose biosynthetic process (GO: 0009298), viral genome replication (GO: 0019079), negative regulation of cysteine-type endopeptidase activity involved in the apoptotic process (GO: 0043154), and regulation of hydrolase activity (GO: 0051336) at the biological process (BP) level. In the category of CC, the upregulated proteins were enriched in the extracellular region (GO: 0005576), an integral component of the membrane (GO: 0016021), while the downregulated proteins were enriched in the extracellular region (GO: 0005615, GO: 0005576, GO: 0044421). For molecular function (MF), the upregulated proteins were related to ribose phosphate diphosphokinase activity (GO: 0004749), acyl-CoA dehydrogenase activity (GO: 0003995), magnesium ion binding (GO: 0000287), and oxidoreductase activity (GO: 0016616) (Supplementary Table 2).

### Overview of metabolome differences

**FIGURE 4:**
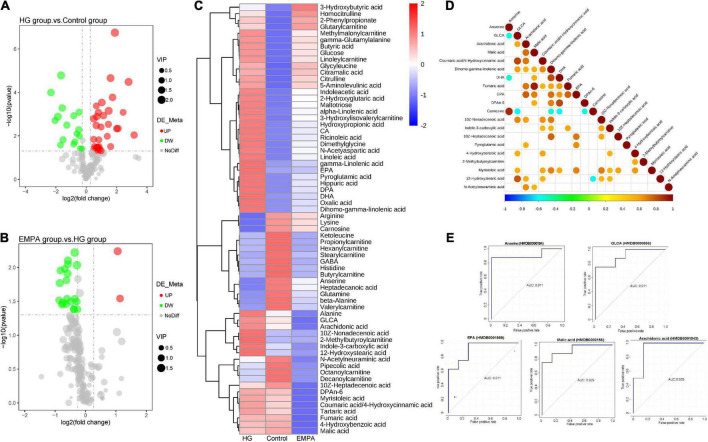
The differential metabolite of the myocardium. **(A,B)** Volcano plots of differential metabolite. **(A)** High glucose (HG) group vs. control group; **(B)** empagliflozin (EMPA) group vs. HG group. Cut-off value: *p* < 0.05, fold change (FC) > 1.2 (upregulation) or < 0.67 (downregulation), the red and green point represents the upregulated and downregulated proteins, respectively. **(C)** Hierarchical clustering analysis of differential metabolite of myocardium in three groups. **(D)** The correlation diagram of differential metabolites in groups EMPA and HG (top 20). The blue point represents a negative correlation (negative value), and the red represents a positive correlation (positive value). **(E)** Receiver operating characteristic (ROC) curves of top 5 metabolites [top 5 areas under the curve (AUC)]. The vertical coordinate is the true positive rate (sensitivity), and the horizontal coordinate is the false positive rate (1—specificity).

The results of the N300 metabolome differences are shown in Figure 4 and Supplementary Table 3. The N300 metabolome is a targeted metabolomic technology for high-throughput absolute quantification of small-molecule metabolites, which can perform absolute quantification of 300 + metabolites in myocardium samples. These metabolites cover multiple metabolic pathways, including the tricarboxylic acid (TCA) cycle, glycolysis/gluconeogenesis, amino acid metabolism, FA synthesis, and bile acid biosynthesis. The detailed detection list of N300 is shown in Supplementary Table 4. A total of 188 metabolites were identified. Of these, 49 metabolites (32 upregulated, 17 downregulated) exhibited differential expression between the control and HG groups, and 22 metabolons (two upregulated and 20 downregulated) exhibited differential expression between the HG and EMPA groups (*p* < 0.05, fold change > 1.2 or < 0.833) (Supplementary Table 3). The majority of differential metabolites exhibited a high correlation with other metabolites (Figure 4D), indicating that the screened-out metabolites may cooperate. The volcano plots and hierarchical clusters visually display the overall distribution of differential metabolites (Figures 4A–C).

The upstream TCA cycle metabolite citric acid was significantly increased, while the downstream metabolites isocitrate, oxoglutaric acid, and succinic acid showed no significant change, and the malic acid content was significantly decreased. This may reflect a relative reduction in TCA turnover, leading to the accumulation of metabolic intermediates and insufficient ATP production in the diabetic heart. Alpha-linolenic acid, gamma-linolenic acid, linoleic acid, and dihomogamma-linolenic acid were significantly increased in the rat’s heart in the HG group, which was associated with biosynthesis of unsaturated FAs and linoleic acid metabolism. Beta-alanine, GABA, and anserine related to beta-alanine metabolism were downregulated in the HG group. In summary, the metabolome data of the control and HG groups suggested that FA metabolism was enhanced in the diabetic heart. Figure 4E shows the ROC curves of metabolites associated with EMPA treatment. Arachidonic acid, malic acid, GLCA, anserine, and EPA were the top five metabolites with the largest area under the curve (AUC), which may be important target metabolites for SGLT2i to exert cardio-protective effects.

### Combined analysis of proteome and metabolome differences

**FIGURE 5:**
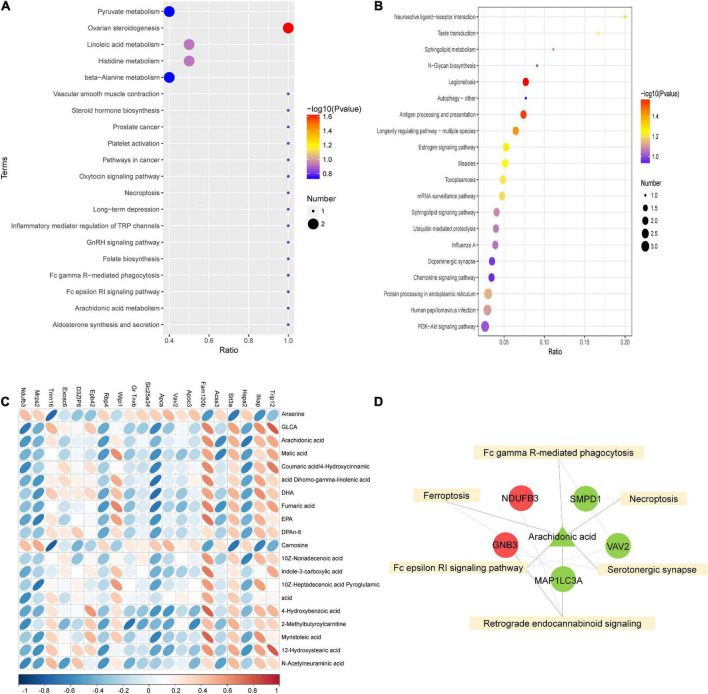
Combined analysis of differential proteins and differential metabolites. **(A,B)** KEGG enrichment bubble diagram of differential proteins **(A)** and differential metabolites **(B)** in groups EMPA and HG. **(C)** Heat map of correlation between differential protein and differential metabolite expression. The horizontal axis represents proteins, and the vertical axis represents metabolites. **(D)** Combined analysis of pathway enrichment of differential protein and metabolites. The yellow square represents the pathways shared by differential metabolites and differential proteins, and the circles and triangle represent the differential proteins and differential metabolites involved in the pathway, respectively. Circles represent differential proteins, triangles represent differential metabolites, red is represent up-regulated and green is down-regulated.

To further investigate the mechanism of SGLT2 inhibitors in treating DCM, KEGG pathway enrichment of proteome and metabolome differences was performed in the EMPA and HG groups (Figures 5A,B). The results revealed that the Fc epsilon RI signaling pathway (map04664), Fc gamma R-mediated phagocytosis (map04666), ferroptosis (map04216), serotonergic synapse (map04726), necroptosis (map04217), and retrograde endocannabinoid signaling (map04723) were common enrichment pathways for differential metabolites and proteins (Figure 5D). The upregulated proteins Ndufb3 and Gnb3, the downregulated proteins Smpd1, Vav2, and Map1lc3a, and the downregulated metabolite arachidonic acid were repeatedly enriched in multiple related items. They could be associated with the pathogenesis of EMPA treatment (Figure 5D).

As shown in Figure 5C, Spearman’s correlation analysis was conducted on the 20 most differentially expressed proteins and metabolites to identify the protein-metabolites relationship after EMPA treatment. Strong correlations (*p* < 0.05) were observed in 82 protein-metabolite pairs (Figure 5C). Various metabolites, such as arachidonic acid, were significantly decreased after EMPA treatment and showed a strong positive correlation with Trip12 and Fam120b and a strong negative correlation with Hspa2 and Acss3. The increased metabolites anserine and carnosine after EMPA treatment were negatively correlated with Ilkap, Stt3a, and Trim16 (Supplementary Table 5).

## Discussion

As novel hypoglycemics, SGLT2 inhibitors (SGLT2i) have recently received tremendous attention. The cardiovascular benefits of SGLT2i may be multifactorial and beyond glycemic control (10). Significant reductions in major adverse cardiovascular events (MACEs) have been observed in various large-scale clinical trials (26–28), prompting investigators to continue exploring the mechanism of action of SGLT2i.

In this present study, we established the T1DM rat model by intraperitoneal injection of STZ and treatment with EMPA for 12 weeks to investigate changes in proteins and metabolites in the myocardium. The animal experiment results confirmed that diabetes contributed to a pronounced DCM characterized by mitochondrial dysfunction, impaired lipid metabolism, myocardial fibrosis, and associated diastolic and systolic functional impairments of the heart, which were consistent with the observations in previous studies (21, 29). Dysregulation of metabolism increased production of AGEs and apoptosis, inflammation, and impaired calcium were all suggested to explain the cardiac impairment in DCM (30). Mitochondria play a central role in energy metabolism. More than 95% of ATP is provided by mitochondrial oxidative phosphorylation, and its morphology is linked to energy metabolism. Numerous studies have shown that changes in mitochondrial morphology reflect alterations in energy metabolism. TEM in this study revealed that the mitochondria in the rat cardiomyocytes of the HG group were irregular and disordered. Studies in patients have demonstrated that mitochondrial dysfunction is linked to ventricular hypertrophy and fibrosis (31). A previous study suggested that SGLT2i may improve mitochondrial function and reduce oxygen-reactive stress (32). The present study found that the mitochondrial morphology changes and myocardial fibrosis were ameliorated in the rat cardiomyocytes of the EMPA group, which confirmed the cardiovascular benefit of SGLT2i. Moreover, the results of metabolomics and proteomics in myocardium uncovered numerous differential proteins and metabolites indicative of energy metabolism changes during the diabetic state and after the treatment of EMPA.

HF is preceded by abnormalities in myocardial energy metabolism (33). The present study found that the cardiometabolic alterations of diabetes are mainly independent of plasma FA/lipids, which indicates that only focusing on the lipid level in peripheral blood is insufficient to objectively reflect the actual metabolism of the myocardium. Our proteomics data suggest that FA metabolism is enhanced in the diabetic heart. Proteins involved in the lipid metabolic process [such as acyl-coenzyme A oxidase, peroxisomal bifunctional enzyme, trifunctional enzyme subunit alpha (mitochondrial), malonyl-CoA decarboxylase (mitochondrial), and inositol-1-monophosphatase] were upregulated in diabetic hearts. Under normal conditions, there is very little lipid storage in the myocardium, which depends on the optimal regulation of FA uptake and oxidation. However, in a diabetic heart, the imbalance of FA uptake and utilization promotes the accumulation of lipids and toxic intermediates (such as ceramide and diacylglycerol) in cardiomyocytes, which in turn affects cardiac function (34). Increased intracardiac triglyceride concentrations in diabetic patients are associated with concentric left ventricular remodeling (even in the absence of hypertension), reduced cardiac energy, and reduced peak systolic strain (35). SGLT2i produces unobtrusive changes in plasma lipid levels, typically increases LDL-C and HDL-C levels, and decreases triglyceride levels slightly, probably due to reduced LDL catabolism (36, 37). Similar plasma lipid changes were also observed in the present study. However, our results further showed that lipid accumulation in the cardiomyocytes of diabetic rats was significantly reduced after EMPA treatment, which predicted the improvement of myocardial energy metabolism.

Next, we examined the expression profiles at the protein and metabolic levels and identified 43 proteins and 34 metabolites regulated in the myocardium of diabetic rats by EMPA treatment. The correlation and KEGG enrichment analyses were performed to better understand these proteins and metabolites. The perturbation of biosynthesis of unsaturated FAs and arachidonic acid metabolism were considered responsible for the EMPA treatment’s effect. In the myocardium of diabetic rats treated with EMPA, the level of arachidonic acid was significantly decreased and correlated with many differential proteins, suggesting that it may play an important role in the cardiovascular benefit of EMPA. Arachidonic acid (AA) is an important FA that is metabolized into several bioactive compounds by cyclooxygenases, lipoxygenases, and P450 enzymes, which play an important role in the cardiovascular system (38). Studies have revealed that the metabolites of arachidonic acid play a role in enhancing cardiac dysfunction in diabetic rats following ischemia/reperfusion injury (39) and in the development and progression of cardiac hypertrophy (40). Moreover, the pathway analysis revealed that the pathways, such as the Fc epsilon RI signaling pathway, Fc gamma R-mediated phagocytosis, ferroptosis, serotonergic synapse, necroptosis, and retrograde endocannabinoid signaling, were involved in the mechanism of EMPA treatment. Previous studies have found that increased apoptosis is implicated in several diabetic complications and plays an important role in the progression of DCM (41, 42). Ferroptosis is an iron-dependent regulated cell death characterized by lipid peroxidation and iron overload, which is morphologically, biochemically, and genetically different from other types of programmed cell death (43). The occurrence and execution of ferroptosis are regulated by amino acids, lipids, and iron metabolism (44). Hence, we speculated that EMPA reversed high glucose-induced cardiomyocyte injury by ameliorating cardiomyocyte apoptosis, ferroptosis, and abnormal metabolism. In addition, enrichment analysis revealed some novel dysregulated signaling pathways, such as the Fc epsilon RI signaling pathway and the retrograde endocannabinoid signaling pathway, whose functions need further investigation.

In summary, metabolic disorders play a major role in the pathogenesis of DCM. The present study confirmed that SGLT2i treatment ameliorated lipid accumulation and mitochondrial damage in the myocardium of diabetic rats. Metabolomic and proteomic analyses in the myocardium of EMPA-treated diabetic rats identified the potential targets and signaling pathways related to the cardiovascular benefit of SGLT2i, which provides a valuable resource for comparative studies.

## Data availability statement

The proteomics data presented in this study are deposited in the ProteomeXchange repository, accession number: PXD036090.

## Ethics statement

This animal study was reviewed and approved by the Medical Ethics Committee of the Dongguan Affiliated Hospital of Jinan University.

## Author contributions

YX, DC, JX, and JG conceived and designed the experiments. YX wrote the main manuscript text. ZD, HL, JH, JZ, YX, CC, and KD performed the experiments. YX and ZD analyzed the data. All authors reviewed the manuscript.
